# Nitric oxide synthase modulates CFA-induced thermal hyperalgesia through cytokine regulation in mice

**DOI:** 10.1186/1744-8069-6-13

**Published:** 2010-03-02

**Authors:** Yong Chen, Michael K Boettger, Andreas Reif, Angelika Schmitt, Nurcan Üçeyler, Claudia Sommer

**Affiliations:** 1 Department of Neurology, University of Würzburg, Josef-Schneider-Str 11, 97080 Würzburg, Germany; 2 Department of Psychiatry and Psychotherapy, University of Würzburg, Füchsleinstr 15, 97080 Würzburg, Germany

## Abstract

**Background:**

Although it has been largely demonstrated that nitric oxide synthase (NOS), a key enzyme for nitric oxide (NO) production, modulates inflammatory pain, the molecular mechanisms underlying these effects remain to be clarified. Here we asked whether cytokines, which have well-described roles in inflammatory pain, are downstream targets of NO in inflammatory pain and which of the isoforms of NOS are involved in this process.

**Results:**

Intraperitoneal (i.p.) pretreatment with 7-nitroindazole sodium salt (7-NINA, a selective neuronal NOS inhibitor), aminoguanidine hydrochloride (AG, a selective inducible NOS inhibitor), L-N(G)-nitroarginine methyl ester (L-NAME, a non-selective NOS inhibitor), but not L-N(5)-(1-iminoethyl)-ornithine (L-NIO, a selective endothelial NOS inhibitor), significantly attenuated thermal hyperalgesia induced by intraplantar (i.pl.) injection of complete Freund's adjuvant (CFA). Real-time reverse transcription-polymerase chain reaction (RT-PCR) revealed a significant increase of nNOS, iNOS, and eNOS gene expression, as well as tumor necrosis factor-alpha (TNF), interleukin-1 beta (IL-1β), and interleukin-10 (IL-10) gene expression in plantar skin, following CFA. Pretreatment with the NOS inhibitors prevented the CFA-induced increase of the pro-inflammatory cytokines TNF and IL-1β. The increase of the anti-inflammatory cytokine IL-10 was augmented in mice pretreated with 7-NINA or L-NAME, but reduced in mice receiving AG or L-NIO. NNOS-, iNOS- or eNOS-knockout (KO) mice had lower gene expression of TNF, IL-1β, and IL-10 following CFA, overall corroborating the inhibitor data.

**Conclusion:**

These findings lead us to propose that inhibition of NOS modulates inflammatory thermal hyperalgesia by regulating cytokine expression.

## Background

Several lines of evidence indicate a role for nitric oxide (NO) as a mediator of inflammation [1,2]. NO, acting as an inter- and intracellular messenger molecule in the peripheral and central nervous system, also plays a pivotal role in the development and maintenance of hyperalgesia [3-6]. NO can be synthesized by three well-characterized isoforms of NO synthase (NOS): the constitutive neuronal NOS (nNOS), endothelial NOS (eNOS), and the inducible NOS (iNOS) [7-9]. The non-selective NOS inhibitor L-N(G)-nitroarginine methyl ester (L-NAME) reduces thermal hyperalgesia in inflammatory pain models [10-12]. Further studies suggested beneficial effects of the selective NOS inhibitors in reducing inflammatory hyperalgesia, while the baseline nociceptive responses remained unaltered [11,13-18].

Inflammatory pain hypersensitivity is the consequence of alterations in transduction sensitivity of high threshold nociceptors [19], activity-dependent changes in the excitability of spinal neurons [20], and phenotypic changes in sensory neurons innervating the inflamed tissue [21]. These changes, both at the inflamed site and throughout the nervous system, are initiated by a complex pattern of chemical signals interacting with the sensory fiber terminals. These signals originate from infective agents, damaged host cells or activated immune cells. Pro- and anti-inflammatory cytokines are small regulatory proteins that are produced by white blood cells and a variety of other cells including those in the nervous system. Inflammatory stimuli or tissue injuries stimulate the release of cytokines, which play an essential role in inflammatory pain. Pro-inflammatory cytokines, such as tumor necrosis factor (TNF) and interleukin-1 beta (IL-1β), reduced thermal or mechanical pain thresholds upon intraplantar application [22-24]. Pro-inflammatory cytokine antagonists were further able to reduce hyperalgesia in inflammation models, indicating that the activation of pro-inflammatory cytokines is an important step in the generation of inflammatory pain [24,25]. To limit the deleterious consequences of prolonged action of pro-inflammatory cytokines, their release is followed by the release of anti-inflammatory cytokines, such as IL-4, IL-10, and IL-13, which inhibit the production and action of the pro-inflammatory cytokines and are anti-hyperalgesic [24]. Correlations between tissue levels of cytokines and pain and hyperalgesia have been described in a number of painful states [26,27]. Although cytokines have well-described roles in inflammatory pain, it is poorly understood what regulates their production and release.

It has been largely demonstrated that inhibition of NOS attenuates inflammatory pain [11,13-18], however, the molecular mechanisms underlying these effects remain to be clarified. NO is generated in significant concentrations at sites of inflammation in which multiple hyperalgesic inflammatory mediators, such as cytokines, prostaglandin E2 (PGE2), or serotonin, are also produced [3,28]. NO may facilitate the hyperalgesia induced by those mediators using the cAMP second messenger pathway and may also have an independent cGMP-dependent hyperalgesic effect. The literature pre-dominantly documents that pro-inflammatory cytokines stimulate the production of NO, suggesting that cytokines modulate pain by regulating the release of NO [28-34]. In contrast, the effect of NO on pro-inflammatory cytokines has rarely been examined. One study reported that human immunodeficiency virus-1 (HIV-1) envelope glycoprotein gp120 stimulates pro-inflammatory cytokine-mediated pain facilitation via activation of nNOS [35]. This finding raises the intriguing possibility that reduction of inflammatory hyperalgesia with NOS inhibitors may be caused, at least in part, by reduced production of pro-inflammatory cytokines. This led us to hypothesize that cytokines, including pro- and anti-inflammatory cytokines, may be involved in pain modulation by NOS under inflammatory conditions. Here, we used a complete Freund's adjuvant (CFA)-induced inflammatory pain model in mice, to investigate whether the expression of cytokines is involved in the NOS-mediated inflammatory thermal hyperalgesia.

## Results

### Pretreatment with NOS inhibitors attenuates CFA-induced thermal hyperalgesia

**Figure 1:**
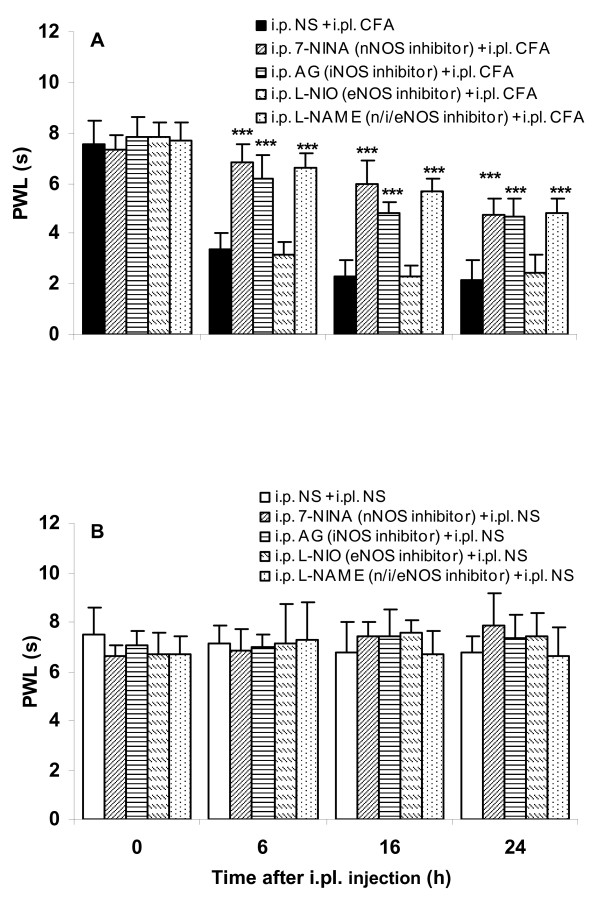
**Effects of pretreatment with the NOS inhibitors 7-NINA, AG, L-NIO and L-NAME on CFA-induced thermal hyperalgesia assessed by the paw withdrawal latency (PWL) tests**. Following CFA injection, PWLs were markedly decreased within 6 h and continued until 24 h post injection in the ipsilateral hindpaw (A, ***P < 0.001, NS + CFA vs NS + NS). Pretreatment with 7-NINA, AG and L-NAME, but not L-NIO, dramatically attenuated thermal hyperalgesia in mice receiving CFA throughout the observation period (A, ^###^*P *< 0.001, inhibitor + CFA vs NS + CFA). None of the inhibitors altered pain thresholds in mice receiving NS (B, inhibitor + NS vs NS + NS). n = 4 for each group.

Thermal pain thresholds were not different between groups at baseline, and not significantly changed after NS injections (Fig. 1). At 6, 16, and 24 h after i.pl. injection of CFA, significant thermal hyperalgesia was observed on the injected side (Fig. 1A).

Preemptive administration of 7-NINA, AG, or L-NAME, but not L-NIO, at a dose of 50 mg/kg, dramatically attenuated CFA-induced thermal hyperalgesia at 6, 16, and 24 h after injection (Fig 1A, *P *< 0.001). In mice receiving NS, none of the inhibitors affected pain thresholds throughout the observation period (Fig. 1B).

### CFA increases both NOS and cytokine gene expression in plantar skin

**Figure 2:**
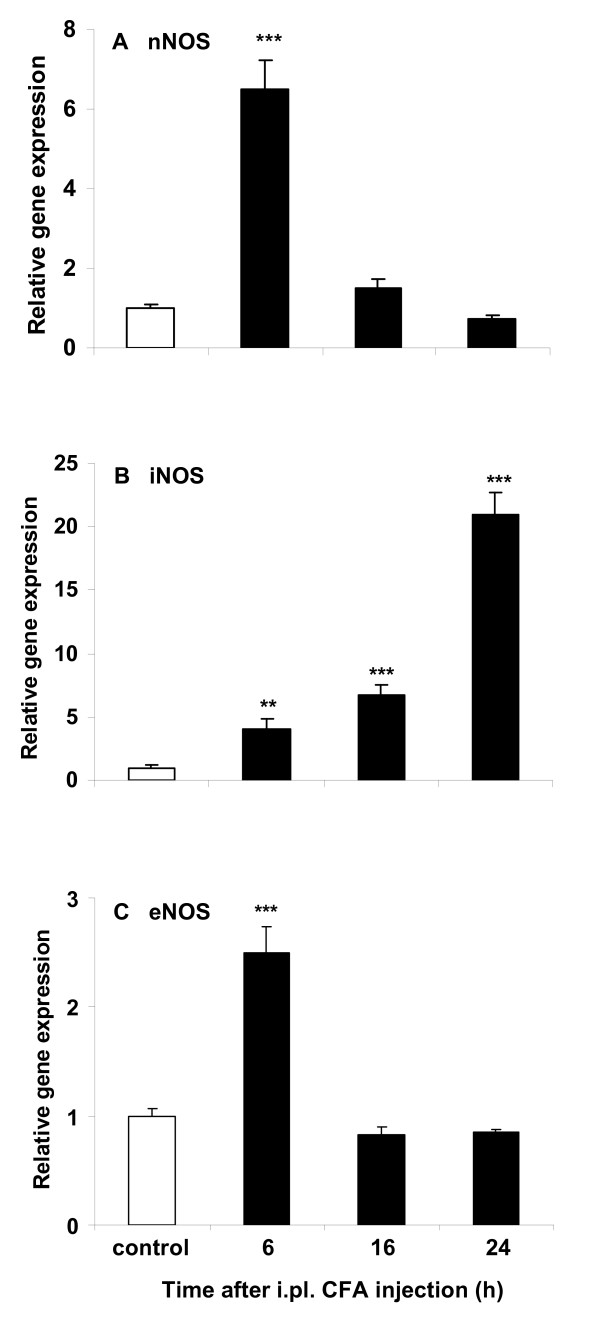
**Relative gene expression of nNOS (A), iNOS (B) and eNOS (C) in the ipsilateral plantar skin in mice receiving CFA compared to control mice receiving NS (NS + CFA vs NS + NS)**. NNOS (A) and eNOS (C) gene expression was increased at 6 h (***P < 0.001), followed by a rapid decline to baseline levels at 16 and 24 h, after CFA. INOS gene expression was increased at 6 h, and peaked at 24 h, after CFA (B, **P < 0.01 and ***P < 0.001). At each time point n = 4.

The gene expression of nNOS (Fig. 2A) and eNOS (Fig. 2C) was elevated in the ipsilateral plantar skin at 6 h after CFA (*P *< 0.001), followed by a rapid decline to baseline levels at 16 and 24 h, compared to controls. INOS gene expression was increased at 6 h and peaked at 24 h after CFA (Fig. 2B, *P *< 0.01 and *P *< 0.001).

**Figure 3:**
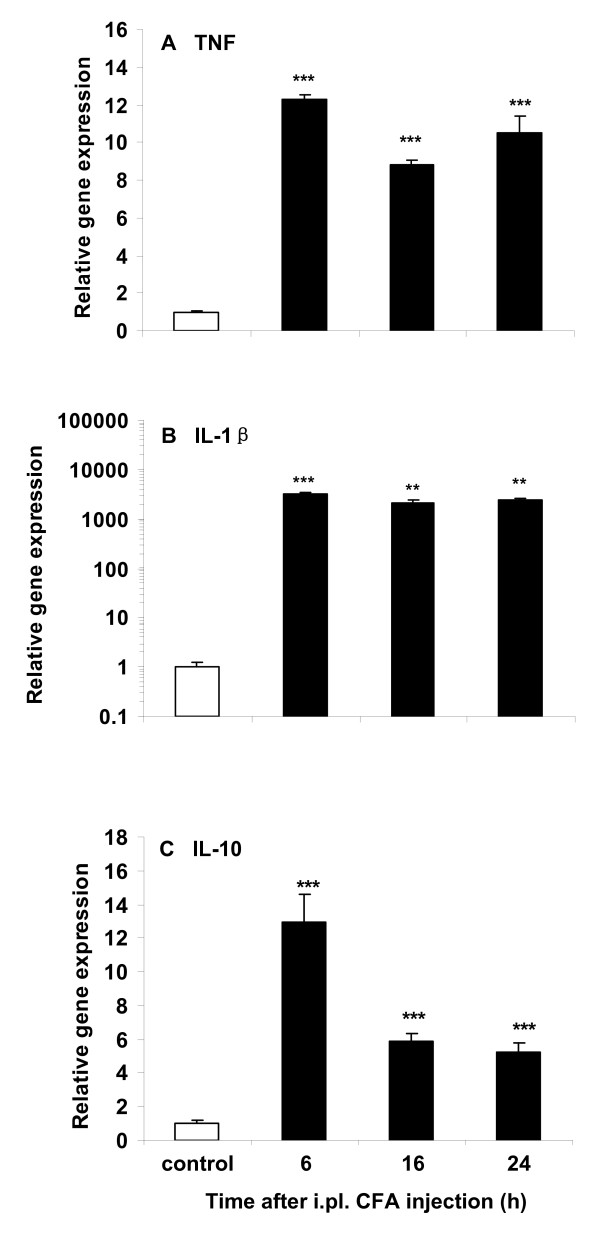
**Relative gene expression of TNF (A), IL-1β (B) and IL-10 (C) in the ipsilateral plantar skin in mice receiving CFA compared to control mice receiving NS (NS + CFA vs NS + NS)**. Note the rapid increase at 6 h, followed by a second rise for TNF and IL-1β at 24 h and a decline for IL-10 at 16 and 24 h, after CFA (**P < 0.01 and ***P < 0.001). At each time point n = 4.

As early as 6 h after CFA, TNF (Fig. 3A), IL-1β (Fig. 3B), and IL-10 (Fig. 3C) gene expression in plantar skin was significantly increased compared to controls (*P *< 0.001) and remained elevated (with a decline for IL-10) until 24 h (*P *< 0.01 and *P *< 0.001). IL-1β mRNA showed the largest increase of expression compared to control (× 2200~3000).

### Pretreatment with the NOS inhibitors reduces the increase of TNF and IL-1β gene expression and has a differential effect on the increase of IL-10 in plantar skin after CFA

**Figure 4:**
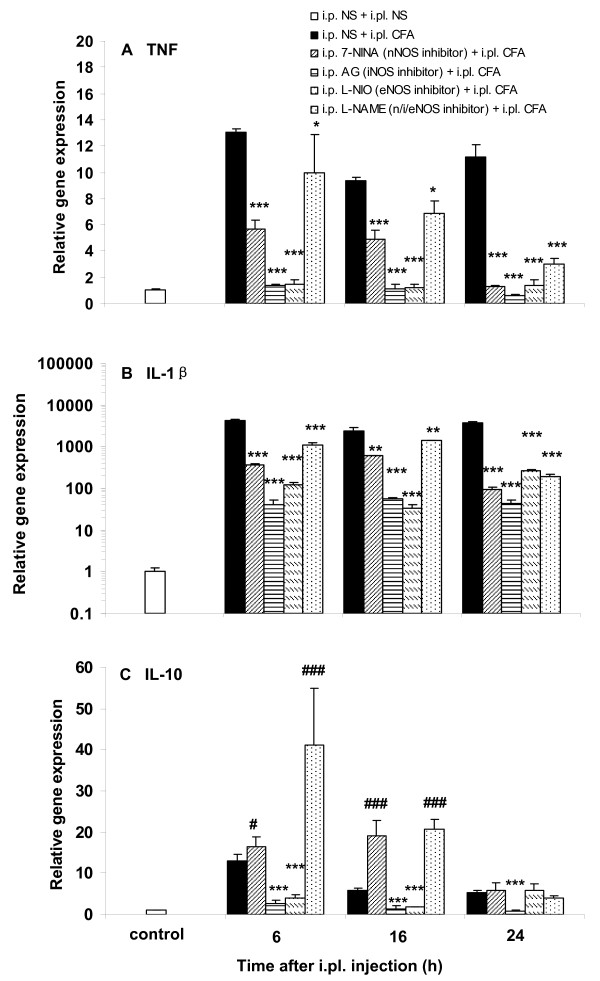
**Effects of pretreatment with the NOS inhibitors 7-NINA, AG, L-NIO and L-NAME on cytokine gene expression after CFA**. All treatments prevented the increase of TNF (A, *P < 0.05 and ***P < 0.001; inhibitor + CFA vs NS + CFA) and IL-1β (B, **P < 0.01 and ***P < 0.001; inhibitor + CFA vs NS + CFA) gene expression induced by CFA. In contrast to the effects of AG and L-NIO, which reduced the increase of IL-10 gene expression at 6, 16, 24 h and at 6 and 16 h (C, ***P < 0.001; AG + CFA or L-NIO + CFA vs NS + CFA), respectively, both 7-NINA and L-NAME at 6 and 16 h significantly enhanced the increase of IL-10 (C, ^#^*P *< 0.05 and ^###^*P *< 0.001; 7-NINA + CFA or L-NAME + CFA vs NS + CFA), after CFA. At each time point n = 4.

Pretreatment with 7-NINA, AG, L-NIO, or L-NAME at a dose of 50 mg/kg did not significantly alter cytokine gene expression in plantar skin of control mice (data not shown). However, all inhibitors significantly attenuated the increase of TNF and IL-1β in mice receiving CFA (Fig. 4A and 4B, *P *< 0.05, *P *< 0.01 and *P *< 0.001). The increase of IL-10 was augmented in mice pretreated with 7-NINA or L-NAME, but reduced in mice receiving AG or L-NIO, at 6 and 16 h after CFA (Fig. 4C, *P *< 0.05 and *P *< 0.001).

### Cytokine gene expression in plantar skin is lower in NOS-KO mice after CFA compared to WT mice

**Figure 5:**
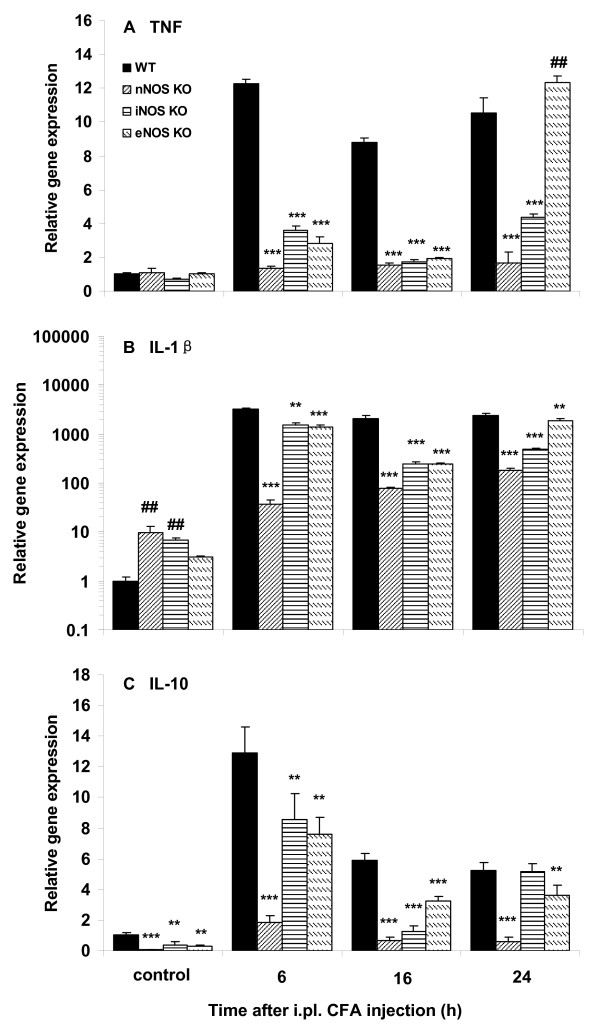
**Cytokine gene expression was lower in NOS-KO mice after CFA compared to WT mice**. In control mice, there was no significant difference in baseline gene expression of TNF between KO and WT (A); IL-1β in nNOS- and iNOS-KO, but not in eNOS-KO, was significantly higher than in WT (B;^##^*P *< 0.01); IL-10 in KO was significantly lower than in WT (C; **P < 0.01 and ***P < 0.001). After CFA, the nNOS-, iNOS- and eNOS-KO mice displayed lower TNF, IL-1β and IL-10 gene expression (A, B and C respectively; ***P < 0.001; nNOS-, iNOS- or eNOS-KO CFA vs WT CFA), except that eNOS-KO mice had a higher TNF when compared to WT mice (A;^###^*P *< 0.001) and there was no difference in IL-10 between iNOS-KO and WT mice (C), at 24 h. All measured samples were related to WT control. At each time point n = 4.

Baseline gene expression of TNF was not different between nNOS-, iNOS- or eNOS-KO mice and WT mice (Fig. 5A). However, the baseline gene expression of IL-1β was significantly higher (Fig. 5B; *P *< 0.01) and that of IL-10 lower (Fig. 5C; *P *< 0.01 and *P *< 0.001) in KO mice than in WT mice, except for IL-1β in eNOS-KO mice, which was not different from WT (Fig. 5B).

After CFA, TNF (Fig. 5A), IL-1β (Fig. 5B), and IL-10 (Fig. 5C) gene expression in nNOS-, iNOS- or eNOS-KO mice was lower than in WT mice at all time points (*P *< 0.01 and *P *< 0.001), except for TNF in eNOS-KO and IL-10 in iNOS-KO mice at 24 h, where they were higher (Fig. 5A; *P *< 0.01) and equal (Fig. 5C), respectively.

## Discussion

The present study provided the following major findings: 1) pretreatment with 7-NINA (a selective nNOS inhibitor), AG (a selective iNOS inhibitor), or L-NAME (a non-selective NOS inhibitor), but not L-NIO (a selective eNOS inhibitor), attenuated CFA-induced thermal hyperalgesia in mice; 2) CFA caused an increase of nNOS, iNOS and eNOS, as well as of TNF, IL-1β, and IL-10 gene expression in plantar skin; 3) preemptive systemic administration of the NOS inhibitors reduced CFA-induced increase of TNF and IL-1β, and produced differential effects on IL-10; 4) following CFA, nNOS-, iNOS- or eNOS-knockout (KO) mice had lower gene expression of TNF and IL-1β, in accordance with the inhibitor data. Together, the findings imply that NOS mediates inflammatory thermal hyperalgesia by regulating cytokine expression.

Significant evidence links NO with the development and maintenance of inflammatory pain (see introduction). In addition, NO has been described either as pro- or anti-inflammatory and can produce both pro- and anti-nociceptive effects. The dual effects of NO in inflammatory pain may be related to the testing paradigms, pain models, doses of NO donors and inhibitors, etc [4]. In the present study, preemptive administration of 7-NINA, AG, or L-NAME, at a dose of 50 mg/kg, reduced thermal hyperalgesia caused by CFA, in line with recent studies [10,13,15]. Compared to the anti-hyperalgesic effects of 7-NINA and AG, we found that L-NIO, a selective eNOS inhibitor, had no significant effect on thermal hyperalgesia. This is in accordance with the finding by Tao et al. [18] who found that L-NIO did not affect carrageenan-induced thermal hyperalgesia in mice.

NO may activate sensory fibers directly, and may influence afferent fiber sensitivity indirectly by altering the availability of mediators such as calcitonin gene-related peptide (CGRP) and substance P (SP) [43]. In various pain models, increased expression of one or more of the three NOS isoforms has been shown in the spinal cord of rodents after i.pl. injection of capsaicin [44], formalin [45], carrageenan [17], or CFA [10,40]. However, to our knowledge, the expression of NOS in peripheral tissues is poorly explored. In the present study, iNOS gene expression in plantar skin was upregulated after CFA, in line with the report by De Alba et al. [13] who found that iNOS immunoreactivity in inflamed paw tissue was increased after CFA. We have extended these findings and have shown for the first time that CFA injection also increased the nNOS and eNOS gene expression. Together, these studies indicate that NOS can be induced not only in the central nervous system but also in the peripheral inflamed tissues after CFA, indicating a role in central and peripheral sensitization in inflammatory pain.

Proinflammatory cytokines like TNF and IL-1β induce and facilitate neuropathic as well as inflammatory pain [26,46-50]. On the other hand, the anti-inflammatory cytokine IL-10, which can suppress pro-inflammatory cytokine production, exhibits antinociceptive effects in different pain models [51-54]. Both pro- and anti-inflammatory cytokines can be released by local or migrating cells, and their balance modulates pain sensitivity. We found that CFA injection resulted in a significant elevation of TNF and IL-1β gene expression in the inflamed plantar skin, which is in accordance with the change of TNF and IL-1β at the protein level in the same tissue [47,49]. In addition, we detected for the first time that CFA also induced an upregulation of IL-10. Following CFA, mice pretreated with 7-NINA, AG, L-NIO or L-NAME had a reduced increase of TNF and IL-1β. CFA-induced upregulation of IL-10 was increased in 7-NINA pretreated mice, but attenuated in mice receiving AG or L-NIO. Interestingly, pretreatment with L-NAME, a non-selective NOS inhibitor, augmented the increase of IL-10 in CFA mice. L-NAME has a higher selectivity and potency for nNOS than for eNOS and iNOS [55-58]; therefore, it might be possible that L-NAME preferentially inhibited nNOS, but not eNOS and iNOS, and thus produced the effect on IL-10, like the selective nNOS inhibitor 7-NINA. The exact mechanisms underlying the effects of the NOS inhibitors on cytokine expression are still not clear. NO activates the cyclic adenosine monophosphate (cAMP) cascade by S-nitrosylation activation of adenylate cyclase and by phosphorylation of cAMP response element-binding protein (CREB) through NO-activated cyclic guanosine monophosphate (cGMP). These second messengers can lead to the activation of transcription factors that modulate the expression of TNF, IL-1β, and IL-10 [59-61]. In addition, the NO signaling pathway can mediate the expression of CREB target genes by controlling CREB-DNA binding, which is independent of CREB phosphorylation [62].

We then tested whether the situation of altered cytokine expression in mice receiving NOS inhibitors can be mimicked in NOS-KO mice. Following CFA, TNF and IL-1β gene expression in nNOS-, iNOS- or eNOS-KO mice was significantly lower than in WT mice. Our data suggest that both inhibition of NOS (using the selective NOS inhibitors) and disruption of the NOS gene (using knock-out mice) can produce similar effects on TNF and IL-1β gene expression. In a similar pattern, the reduced gene expression of IL-10 was concordant between the iNOS inhibitor AG treated- and iNOS-KO-mice, and the eNOS inhibitor L-NIO treated- and eNOS-KO-mice. Different from mice pretreated with the nNOS inhibitor 7-NINA, which had an augmented IL-10 gene expression, nNOS-KO animals showed lower expression of IL-10. The factors that may account for this discrepancy in IL-10 gene expression between the nNOS inhibitor pretreated mice and the nNOS-KO animals are still unknown. Previous studies indicated that eNOS and iNOS can compensate for the function of nNOS in nNOS-KO mice in the carrageenan [18] and CFA [63] models of inflammation, respectively. We speculate that nNOS may be compensated by iNOS and eNOS in nNOS-KO mice here, which may lead to a lower gene expression of IL-10.

Considering that TNF and IL-1β contribute to the generation of inflammatory pain and that IL-10 has anti-hyperalgesic effects, it is conceivable that both the attenuation of the increase of TNF and IL-1β and an augmentation of the increase of IL-10 by 7-NINA and L-NAME may contribute to the mechanism by which pretreatment with 7-NINA and L-NAME reduces CFA-induced thermal hyperalgesia. In the same model, however, changes in hyperalgesia were absent [10] or less pronounced [63] in nNOS-KO mice. One possible explanation for these differences is that disruption of the nNOS gene not only reduces TNF and IL-1β, but also decreases IL-10. Similarly, CFA-caused thermal hyperalgesia was not significantly altered in mice either receiving the eNOS inhibitor L-NIO (in the present study) or disrupting the eNOS gene [63] because the hyperalgesic TNF, IL-1β and the anti-hyperalgesic IL-10 were simultaneously reduced for both treatments. Interestingly, compared to the unchanged hyperalgesia in the eNOS inhibitor L-NIO-treated mice after CFA, thermal hyperalgesia was significantly attenuated by the iNOS inhibitor AG, although AG also simultaneously reduced TNF, IL-1β and IL-10. Different from what we observed in the iNOS inhibitor AG-treated mice, the attenuation of CFA-induced thermal hyperalgesia was less pronounced in iNOS-KO mice [63]. The mechanisms underlying these paradoxical phenomena are still unknown. Inflammation triggers a bi-directional activation of neurons and immune cells with subsequent balanced release of both pro-inflammatory and anti-inflammatory cytokines which can act locally or at a distance. Loss of this pro- and anti-inflammatory balance may underlie different pain states [27]. Differences in this balance in mice receiving different NOS inhibitors or in mice lacking different NOS genes may underlie the differential effects on behavior. Similar discrepancies in pain behavior between NOS inhibitor-treated mice and NOS-KO mice have been observed by others, and are conceivable given the probably large amount of uncontrolled compensatory changes in KO mice [10,17,18]. Last, our study does not exclude the possibility that other cytokines or chemokines and other pain mediators, such as bradykinin or prostaglandins, play a role in the modulation of inflammatory pain by NOS.

## Conclusion

In summary, several lines of evidence indicate that cytokines regulation is a novel mechanism by which inhibition of NOS modulates CFA-induced inflammatory thermal hyperalgesia. Furthermore, our present data and studies from other groups [28-34] suggest that there might be a feedback loop between NO and cytokines which modulates inflammatory pain.

## Methods

### Animals

Experiments were performed on adult (4-7 months, 25-30 g body weight) male mice of C57BL/6J background. These included wild type mice, mice deficient for iNOS [36], eNOS [37], and mice deficient for nNOS of 129S4BL/6J background (The Jackson Laboratory, Bar Harbor, Maine, USA) [38]. All mice were bred at the animal facilities of the University of Würzburg. The animals were housed on a light:dark cycle of 14:10 h with standard rodent chow and water available *ad libitum*. All experiments were approved by the Bavarian state authorities and performed in accordance with the European Communities Council Directive of November 24, 1986 (86/609/EEC) for the care and use of laboratory animals.

### Drugs and drug administration

Intraplantar (i.pl.) injections were performed with a Hamilton syringe coupled to a 30-gauge needle under light ether anesthesia. Control mice received 0.5 ml of normal saline (NS) by intraperitoneal (i.p.) injection, and, 30 min later, 10 μl of NS intraplantarly into the surface of one hind paw (control group: NS + NS). For induction of hindpaw inflammation, mice received i.pl. injection of 10 μl of complete Freund's adjuvant (CFA, diluted 1:1 with PBS, 2 mg/ml; Mycobacterium tuberculosis; Difco Laboratories, Detroit, MI) 30 min after the i.p. NS injection (CFA group: NS + CFA). For the inhibitor experiments, the inhibitors including 7-nitroindazole sodium salt (7-NINA, a selective nNOS inhibitor; A.G. Scientific, Inc. Göttingen, Germany), aminoguanidine hydrochloride (AG, a selective iNOS inhibitor; Sigma, Munich, Germany), L-N(5)-(1-iminoethyl)-ornithine (L-NIO, a selective eNOS inhibitor; Biotium Inc., Hayward, CA) and L-N(G)-nitroarginine methyl ester (L-NAME, a non-selective NOS inhibitor; Sigma) dissolved in NS except for 7-NINA in 20% DMSO, were i.p. injected at a dose of 50 mg/kg 30 min prior to i.pl. injection of CFA or NS (inhibitor pretreated CFA and control groups: inhibitor + CFA and inhibitor + NS).

Mice deficient for nNOS, iNOS and eNOS (KO mice) were i.p. injected with 0.5 ml of NS 30 min before i.pl. administration of 10 μl of CFA or NS (NOS-KO CFA and control groups).

### Behavioral testing

Sensitivity to noxious heat was assessed using the device of Hargreaves et al. [39] purchased from Ugo Basile (Comerio, Italy). A radiant heat source was focused on the plantar surface of the hindpaw; the latency from the initiation of the radiant heat until paw withdrawal (paw withdrawal latency, PWL) was measured automatically. A maximal cutoff of 20 s was used to prevent tissue damage. The injected paw was tested two times; the mean withdrawal latency was calculated. The interval between two trials on the same paw was at least 3 min. Mice were tested 1 d before i.pl. injection of CFA or NS to determine baseline thresholds, and then at 6, 16 and 24 h after injection.

### Quantitative real-time PCR

Plantar skin of the hindpaw was removed 24 h after behavioral testing from control, CFA, and inhibitor pretreated CFA and control groups. In separate mice without behavioral testing, including CFA and inhibitor pretreated CFA groups, plantar skin was harvested at 6 and 16 h after injection. In addition, the same tissue was also dissected out from nNOS-, iNOS-, and eNOS-KO mice at 24 h after NS injection (NOS-KO control groups), and at 6, 16 and 24 h after CFA (NOS-KO CFA groups). At each time point, specimens from one treatment group were pooled (n = 4), immediately shock frozen in liquid nitrogen and stored at -80°C before further processing. Tissue homogenization and RNA isolation were performed as described previously [40]. The frozen tissue was incubated in TRIzol reagent^® ^(Invitrogen, Karlsruhe, Germany) and homogenized with a Polytron homogenizer (Kinematica, Luzern, Switzerland). Afterwards chloroform was added and the samples were centrifuged at 13,000 *g *and 4°C for 15 min. Then, the upper phase was mixed with glycogen and propanol. After incubation over night at -20°C the samples were washed with 75% ethanol and the extracted RNA was dissolved in 33 μl of diethylpyrocarbonate (DEPC) treated water. The total RNA yield was photometrically quantified (Eppendorf, Hamburg, Germany).

Relative NOS and cytokine mRNA expressions were quantified with real-time PCR using the fluorescent TaqMan technology. 500 ng of total RNA were reverse transcribed (TaqMan Reverse Transcription Reagents, Applied Biosystems, Germany) using random hexamers. PCR primers and probes specific for mouse nNOS (Assay-ID: Mm00435175_m1), iNOS (Mm00440485_m1), eNOS (Mm00435204_m1), TNF (Mm00443258_m1), IL-1β (Mm00434228_m1), IL-10 (Mm00439616_m1) and 18s rRNA were obtained from TaqMan Predeveloped Assay Reagents for gene expression (Applied Biosystems, Germany). 18s rRNA was used as an endogenous control. PCR was performed with equal amounts of cDNA in the GeneAmp 7700 sequence detection system (Applied Biosystems, Germany) using TaqMan Universal PCR Master Mix (Applied Biosystems). Reactions (total volume 50 μl) were incubated at 50°C for 2 min, at 95°C for 10 min followed by 40 cycles of 15 s at 95°C and 1 min at 60°C. The comparative Ct method was used for relative quantification of gene expression. The amount of NOS and cytokine mRNAs, normalized to the endogenous control (18s rRNA) and relative to a calibrator (tissue from wild-type (WT) control animals), is given by 2^-ΔΔCt^, with Ct indicating the cycle number at which the fluorescence signal of the PCR product crosses an arbitrary threshold set within the exponential phase of the PCR, and ΔΔCt = [(Ct_target (unknownsample) _- Ct_end.control (unknownsample)_)] - [(Ct_target (calibratorsample) _- Ct_end.control (calibratorsample)_)]. The absolute value of the calibrator was set one and all measured samples were related to this sample. In order to guarantee primer specificity and to exclude genomic contamination, negative controls without cDNA template were run on each RT-PCR well plate.

To test the quality of the total RNA, the samples were photometrically quantified (Eppendorf, Hamburg, Germany) and the 260/280 ratio was measured for the integrity of the extracted RNA. In addition, agarose gel electrophesis of the housekeeping gene glyceraldehyde-3-phosphate dehydrogenase (GAPDH) PCR product was analyzed. PCR amplification of GAPDH was performed at 94°C for 5 min and 35 cycles of 94°C for 45 sec, 58°C for 45 sec and 72°C for 45 sec, followed by 72°C for 10 min, using sequence specific primers (Sigma, Munich, Germany) 5'-TCACCACCA TGGAGAAGGCTG-3' (sense) and 5'-CCCTGTT GCTGTAGCCATATTC-3' (antisense). PCR products were loaded on 1% agarose gel containing ethidium bromide, and bands were visualised under UV rays. To assess the kinetics of RT-PCR, TNF primer was selected for RT-PCR amplification with decreasing concentrations of samples (diluted by 1:1, 1:2, 1:4, and 1:6). These data as above are provided in Additional files 1 and 2.

### Data Analysis

For statistical analysis, SPSS software (Version 11.5; Chicago, IL) was used. As described previously [41,42], each sample was measured in triplicate, and data are given as means ± SD. The data were analyzed by one-way analysis of variance (ANOVA) followed by least significant difference (LSD) *post hoc *test to determine differences between groups. *P *< 0.05 was considered to be statistically significant.

## Competing interests

The authors declare that they have no competing interests.

## Authors' contributions

YC designed and carried out the studies, the data analyses, and drafted the manuscript. MKB and NÜ helped to design the studies and draft the manuscript. AR and AS contributed NOS knockout mice and participated in manuscript editing. CS coordinated and supervised the experiments, analyzed the data and wrote the manuscript. All authors read and approved the final manuscript.

## Supplementary Material

Additional file 1 **Six RNA samples were randomly selected to show the quality of total RNA**. The result of gel electrophoresis using GAPDH primers gave an expected band at 666 bp (A), and the ratio of OD260/OD280 was around 1.9, indicating that the samples were highly purified and largely intact (A and B). Click here for file

Additional file 2 **Six RNA samples and TNF primer were randomly selected to show the kinetics of RT-PCR**. TNF RT-PCR amplification plots with decreasing concentrations of samples suggested that Ct-values increase with further dilutions at 1:1, 1:2, 1:4 and 1:6. Click here for file
