# Supplementary figures and images for: Nitric oxide synthase modulates CFA-induced thermal hyperalgesia through cytokine regulation in mice
Source: Mol Pain. 2010 Mar 2;6:13. doi: 10.1186/1744-8069-6-13 (PMC2838835; doi:10.1186/1744-8069-6-13)

## Slide 1
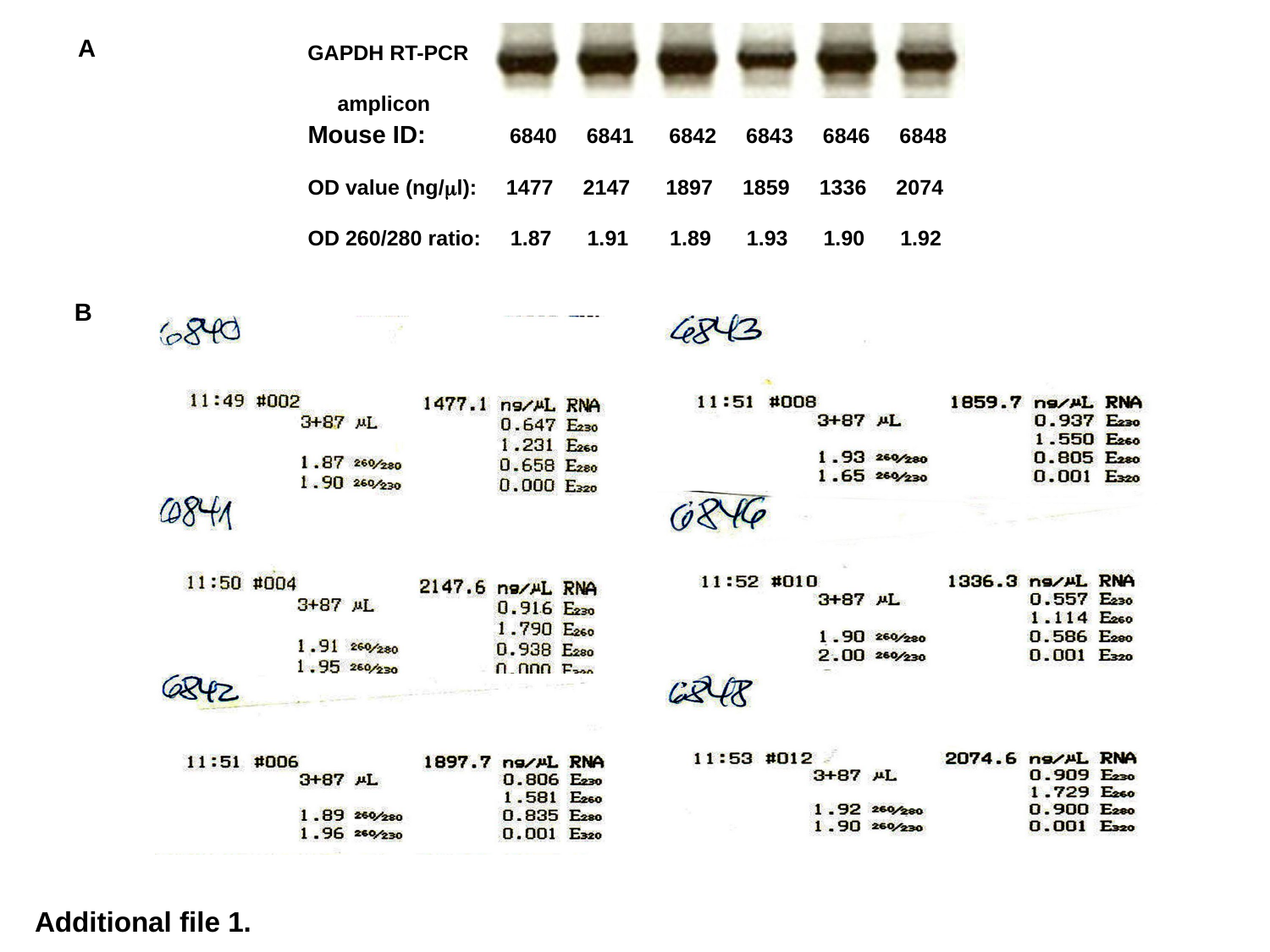

A
GAPDH RT-PCR
 amplicon
Mouse ID: 6840 6841 6842 6843 6846 6848
OD value (ng/μl): 1477 2147 1897 1859 1336 2074
OD 260/280 ratio: 1.87 1.91 1.89 1.93 1.90 1.92
B
Additional file 1.

Supplement: Additional file 1 — Six RNA samples were randomly selected to show the quality of total RNA. The result of gel electrophoresis using GAPDH primers gave an expected band at 666 bp (A), and the ratio of OD260/OD280 was around 1.9, indicating that the samples were highly purified and largely intact (A and B). [file 1744-8069-6-13-S1.PPT]

## Slide 1
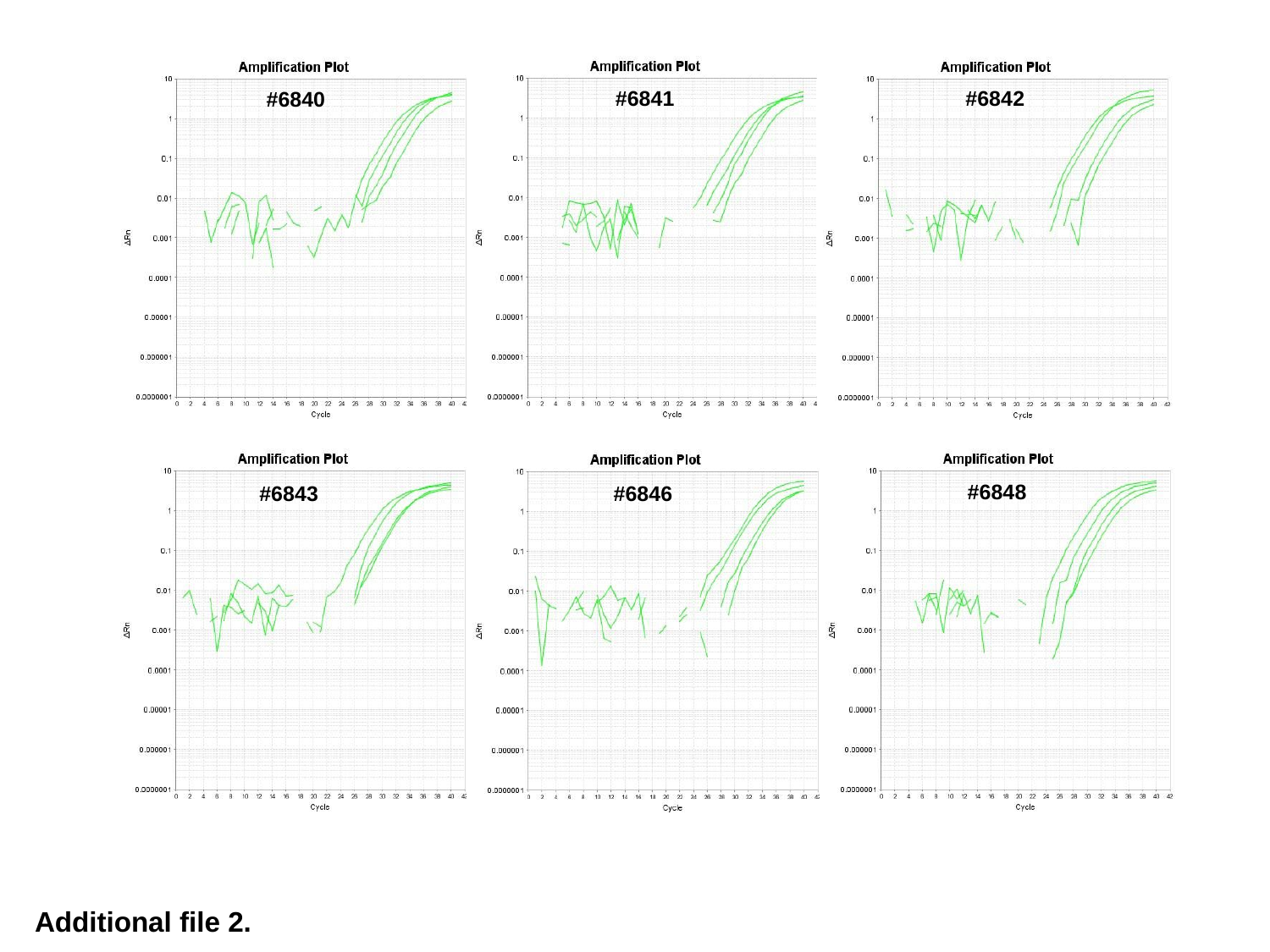

#6841
#6842
#6840
#6848
#6843
#6846
Additional file 2.

Supplement: Additional file 2 — Six RNA samples and TNF primer were randomly selected to show the kinetics of RT-PCR. TNF RT-PCR amplification plots with decreasing concentrations of samples suggested that Ct-values increase with further dilutions at 1:1, 1:2, 1:4 and 1:6. [file 1744-8069-6-13-S2.PPT]
